# Supplementary material for: Meta-Analysis and Gene Set Enrichment Relative to ER Status Reveal Elevated Activity of MYC and E2F in the “Basal” Breast Cancer Subgroup
Source: PLoS One. 2009 Mar 9;4(3):e4710. doi: 10.1371/journal.pone.0004710 (PMC2650420; doi:10.1371/journal.pone.0004710)
Supplement: Dataset S1 — GSEA results from first screen. The file contains a zipped archive of the HTML results of the second GSEA screen. Reports for enriched or depleted datasets may be accessed from the “index.html” link within the my_analysis.GseaPreranked.1219975950625.rpt.zip ZIP archive. Enrichment plots are available for each of the 50 most enriched and depleted gene sets. (13.32 MB ZIP) [file pone.0004710.s001.zip › my_analysis.GseaPreranked.1219975950625.rpt.zip!//CELL_CYCLE_KEGG.html]

Details for gene set CELL\_CYCLE\_KEGG[GSEA]

|  || Dataset | zSco.ERneg.to.ERpos\_collapsed |
| Phenotype | NoPhenotypeAvailable |
| Upregulated in class | na\_pos |
| GeneSet | CELL\_CYCLE\_KEGG |
| Enrichment Score (ES) | 0.6309563 |
| Normalized Enrichment Score (NES) | 2.5002549 |
| Nominal p-value | 0.0 |
| FDR q-value | 0.0 |
| FWER p-Value | 0.0 |
Table: GSEA Results Summary

  

Fig 1: Enrichment plot: CELL\_CYCLE\_KEGG      
 Profile of the Running ES Score & Positions of GeneSet Members on the Rank Ordered List

  

| PROBE | GENE SYMBOL | GENE\_TITLE | RANK IN GENE LIST | RANK METRIC SCORE | RUNNING ES | CORE ENRICHMENT || 1 | E2F3 |  |  | 6 | 7.369 | 0.0302 | Yes |
| 2 | CDC20 |  |  | 9 | 7.151 | 0.0598 | Yes |
| 3 | CCNE1 |  |  | 24 | 6.716 | 0.0867 | Yes |
| 4 | MCM5 |  |  | 31 | 6.533 | 0.1134 | Yes |
| 5 | HDAC2 |  |  | 32 | 6.511 | 0.1405 | Yes |
| 6 | ORC6L |  |  | 33 | 6.507 | 0.1676 | Yes |
| 7 | BUB1 |  |  | 41 | 6.445 | 0.1939 | Yes |
| 8 | CCNA2 |  |  | 52 | 6.297 | 0.2193 | Yes |
| 9 | CDC45L |  |  | 108 | 5.824 | 0.2394 | Yes |
| 10 | CCNA1 |  |  | 117 | 5.732 | 0.2626 | Yes |
| 11 | SKP2 |  |  | 127 | 5.694 | 0.2857 | Yes |
| 12 | MCM4 |  |  | 180 | 5.436 | 0.3043 | Yes |
| 13 | MCM6 |  |  | 198 | 5.317 | 0.3252 | Yes |
| 14 | ORC1L |  |  | 212 | 5.280 | 0.3462 | Yes |
| 15 | PTTG1 |  |  | 225 | 5.206 | 0.3669 | Yes |
| 16 | CHEK1 |  |  | 253 | 5.088 | 0.3860 | Yes |
| 17 | BUB1B |  |  | 271 | 5.016 | 0.4056 | Yes |
| 18 | CDC14B |  |  | 280 | 4.994 | 0.4258 | Yes |
| 19 | CDC25A |  |  | 281 | 4.993 | 0.4466 | Yes |
| 20 | MCM7 |  |  | 289 | 4.970 | 0.4667 | Yes |
| 21 | CDKN2A |  |  | 330 | 4.858 | 0.4839 | Yes |
| 22 | CDC25B |  |  | 340 | 4.826 | 0.5033 | Yes |
| 23 | CCNB2 |  |  | 431 | 4.589 | 0.5156 | Yes |
| 24 | MAD2L1 |  |  | 477 | 4.446 | 0.5307 | Yes |
| 25 | MCM2 |  |  | 630 | 4.080 | 0.5362 | Yes |
| 26 | MCM3 |  |  | 656 | 4.019 | 0.5510 | Yes |
| 27 | TFDP1 |  |  | 750 | 3.843 | 0.5600 | Yes |
| 28 | PTTG3 |  |  | 766 | 3.813 | 0.5747 | Yes |
| 29 | CDC7 |  |  | 769 | 3.807 | 0.5904 | Yes |
| 30 | CHEK2 |  |  | 791 | 3.790 | 0.6046 | Yes |
| 31 | PLK1 |  |  | 988 | 3.499 | 0.6044 | Yes |
| 32 | PRKDC |  |  | 1234 | 3.204 | 0.5992 | Yes |
| 33 | CDC2 |  |  | 1259 | 3.182 | 0.6106 | Yes |
| 34 | PCNA |  |  | 1346 | 3.087 | 0.6170 | Yes |
| 35 | CCNB1 |  |  | 1626 | 2.823 | 0.6077 | Yes |
| 36 | E2F1 |  |  | 1695 | 2.764 | 0.6140 | Yes |
| 37 | CDC25C |  |  | 1794 | 2.700 | 0.6178 | Yes |
| 38 | E2F4 |  |  | 1948 | 2.572 | 0.6170 | Yes |
| 39 | ATM |  |  | 1994 | 2.536 | 0.6241 | Yes |
| 40 | HDAC6 |  |  | 2163 | 2.411 | 0.6215 | Yes |
| 41 | DTX4 |  |  | 2196 | 2.388 | 0.6290 | Yes |
| 42 | CCND2 |  |  | 2506 | 2.197 | 0.6148 | Yes |
| 43 | CDK4 |  |  | 2546 | 2.176 | 0.6209 | Yes |
| 44 | ORC3L |  |  | 2573 | 2.160 | 0.6279 | Yes |
| 45 | CDK2 |  |  | 2688 | 2.099 | 0.6281 | Yes |
| 46 | CCND3 |  |  | 2854 | 2.011 | 0.6240 | Yes |
| 47 | HDAC4 |  |  | 2922 | 1.973 | 0.6271 | Yes |
| 48 | WEE1 |  |  | 3048 | 1.909 | 0.6256 | Yes |
| 49 | E2F5 |  |  | 3105 | 1.885 | 0.6292 | Yes |
| 50 | CDC6 |  |  | 3185 | 1.847 | 0.6310 | Yes |
| 51 | ORC5L |  |  | 3377 | 1.760 | 0.6239 | No |
| 52 | CCNE2 |  |  | 3424 | 1.738 | 0.6276 | No |
| 53 | TBC1D8 |  |  | 4413 | 1.350 | 0.5586 | No |
| 54 | ORC4L |  |  | 4538 | 1.307 | 0.5547 | No |
| 55 | PTTG2 |  |  | 4656 | 1.267 | 0.5511 | No |
| 56 | HDAC1 |  |  | 4997 | 1.157 | 0.5303 | No |
| 57 | E2F2 |  |  | 5382 | 1.019 | 0.5055 | No |
| 58 | TGFB1 |  |  | 5452 | 0.993 | 0.5044 | No |
| 59 | MPL |  |  | 5453 | 0.992 | 0.5085 | No |
| 60 | MDM2 |  |  | 5816 | 0.872 | 0.4848 | No |
| 61 | ESPL1 |  |  | 5874 | 0.853 | 0.4841 | No |
| 62 | CDC14A |  |  | 6609 | 0.618 | 0.4312 | No |
| 63 | ORC2L |  |  | 6907 | 0.517 | 0.4109 | No |
| 64 | TP53 |  |  | 7848 | 0.167 | 0.3406 | No |
| 65 | ABL1 |  |  | 7913 | 0.141 | 0.3364 | No |
| 66 | RB1 |  |  | 8000 | 0.105 | 0.3303 | No |
| 67 | SMAD4 |  |  | 8606 | -0.133 | 0.2852 | No |
| 68 | E2F6 |  |  | 8656 | -0.155 | 0.2821 | No |
| 69 | GADD45A |  |  | 8982 | -0.305 | 0.2589 | No |
| 70 | GSK3B |  |  | 9187 | -0.398 | 0.2451 | No |
| 71 | HDAC5 |  |  | 9206 | -0.408 | 0.2454 | No |
| 72 | MAD1L1 |  |  | 9406 | -0.516 | 0.2326 | No |
| 73 | BUB3 |  |  | 9557 | -0.594 | 0.2237 | No |
| 74 | CDH1 |  |  | 10717 | -1.328 | 0.1417 | No |
| 75 | CDKN1A |  |  | 10787 | -1.380 | 0.1422 | No |
| 76 | HDAC3 |  |  | 10972 | -1.548 | 0.1348 | No |
| 77 | EP300 |  |  | 11100 | -1.663 | 0.1321 | No |
| 78 | HDAC7A |  |  | 11335 | -1.861 | 0.1222 | No |
| 79 | PTPRA |  |  | 11592 | -2.117 | 0.1117 | No |
| 80 | CCNH |  |  | 13015 | -4.513 | 0.0230 | No |
Table: GSEA details [plain text format]

  

Fig 2: CELL\_CYCLE\_KEGG: Random ES distribution      
 Gene set null distribution of ES for **CELL\_CYCLE\_KEGG**
